# Posaconazole oral suspension for secondary antifungal prophylaxis in allogeneic stem cell transplantation recipients: a retrospective study

**DOI:** 10.1186/s12879-022-07442-y

**Published:** 2022-05-15

**Authors:** Peipei Ye, Renzhi Pei, Youqian Hu, Dong Chen, Shuangyue Li, Junjie Cao, Fenglin Li, Mengjie Wu, Ying Fang, Ying Lu

**Affiliations:** Department of Hematology, The Affiliated People’s Hospital of Ningbo University, Ningbo, 315101 China

**Keywords:** Secondary antifungal prophylaxis, Invasive fungal diseases, Posaconazole, Allogeneic hematopoietic stem cell transplantation

## Abstract

**Background:**

There is no consensus on the optimal secondary antifungal prophylaxis (SAP) regimen in patients receiving allogeneic hematopoietic stem cell transplantation (allo-HSCT). The purpose of this study was to evaluate the efficacy and safety of posaconazole oral suspension as secondary prophylaxis of invasive fungal disease (IFD) for allo-HSCT patients.

**Methods:**

We retrospectively reviewed clinical data from prior IFD patients who received posaconazole oral suspension as systemic antifungal prophylaxis between June 2016 and January 2021 and have a follow-up period of 1 year after HSCT. The clinical outcomes of patients with a prior history of IFD (n = 30) and those without (n = 93) were compared.

**Results:**

The 1-year cumulative incidence of prophylaxis failure was 58.3% in the group with prior history of IFD and 41.6% in the group without a prior history of IFD (p = 0.459). The cumulative incidence of proven, probable or possible IFD within 1 year after allo-HSCT was 23.1% in the group with prior history of IFD and 14.1% in the group without prior history of IFD (p = 0.230). There was no significant difference between the cumulative incidence of proven or probable IFD within 1-year after allo-HSCT in the group with a prior history of IFD and the group without (p = 0.807). Multivariate logistic regression revealed cytomegalovirus disease as risk factor for post-transplantation IFD occurrence in posaconazole oral suspension prophylaxis. There was not a significant difference in overall survival between the patients with IFD history and those without (P = 0.559).

**Conclusions:**

Our study support that allo-HSCT recipients with a prior history of IFD and normal GI absorption can choose posaconazole oral suspension as a safe and effective SAP option.

## Introduction

Invasive fungal disease (IFD) is a common infectious complication of allogeneic hematopoietic stem cell transplantation (allo-HSCT) [1]. Especially in China, the incidence of IFD after secondary antifungal prophylaxis (SAP) was 38.6% among patients receiving allo-HSCT [2]. In general, broad-spectrum antifungal agents such as voriconazole, itraconazole, liposomal amphotericin B, and caspofungin are effective for patients with a history of fungal infection [3]. However, due to the lack of large prospective study, there is no consensus on SAP regimen. In order to avoid prophylaxis failure, medication that had been effective and well tolerated in initial antifungal therapy should be used for SAP [4]. However, in study done by Liu, they found no significant difference in IFD occurrence rate between such medication and other broad-spectrum medication. Therefore, using a broad-spectrum antifungal medication as SAP is feasible [3]. In addition, the opportunistic molds and yeast-like fungi (e.g. *Zygomycetes, Fusarium spp.* and *Candida spp*.) are gradually emerging in HSCT recipients during recent broad-spectrum antifungal treatment [5]. Therefore, SAP drug selection needs to take in account of drug resistance from such strains.

Posaconazole has become a widely accepted strategy for IFD prevention in acute myeloid leukemia (AML) or myelodysplastic syndromes (MDS) patients who undergo intensive chemotherapy and allo-HSCT recipients with graft-versus-host disease (GVHD) [6, 7]. Compared with other azole antifungals, it has strengths of broad spectrum, great potency, cost-efficiency and good long-term tolerance [6, 8–11]. However, absorption is still an important factor to consider in efficacy assessment of posaconazole.

In this retrospective study, the efficacy of posaconazole SAP therapy for IFD was assessed. In addition, we compared the clinical outcomes between allo-HSCT recipients with IFD before transplantation and those without when posaconazole was used as systemic antifungal prophylaxis.

## Methods

### Patients

We retrospectively analyzed the records of consecutive adult patients who underwent allogeneic HSCT and used posaconazole as systemic antifungal prophylaxis from June 2016 to January 2021 in The Affiliated People's Hospital of Ningbo University. During this period, 212 patients received allogeneic HSCT. Among the HSCT recipients, 116 (54.7%) received posaconazole oral suspension 200 mg orally three times daily and 7 (3.3%) received posaconazole oral suspension 400 mg twice daily. These 123 patients were included for our study. This study was conducted in accordance with the Declaration of Helsinki and approved by the Ethics Committee of The Affiliated People's Hospital of Ningbo University. All patients have signed informed consent forms to participate in the study.

### Transplant regimens and post-transplant immunosuppression

All patients received conventional regimen such as myeloablative/reduced-intensity conditionings, and were classified based on criteria described by Giralt et al. [12]. Cyclosporine A (CsA) + mycophenolate mofetil (MMF) + a short course of methotrexate (MTX) were administered to the patients undergoing human leukocyte antigen (HLA)-matched sibling donor (MSD) transplant for GVHD prophylaxis. After January 2019, CsA + MMF + MTX + anti-thymocyte globulin (ATG) (ThymoglobulinⓇ, Genzyme, Cambridge, MA) were administered for 1.5 mg/kg i.v. on day-3, -2 and -1 to the MSD transplant patients whose age ≥ 45 years, and CsA + MMF + MTX + ATG (2.5 mg/kg i.v. on day-4, -3, -2 and -1) were administered to the patients undergoing haploidentical donor [13] transplants for GVHD prophylaxis, some haploidentical recipients received transplant cyclophosphamide 50 mg/kg on day + 3.

### Definition

IFD was classified as proven, probable, or possible infection according to the European Organization for Research and Treatment of Cancer and the Mycoses Study Group Education and Research Consortium (EORTC/MSG) [14]. Suspected IFD included these proven, probable, possible IFD. Responses to treatment were classified into CR, partial response (PR), stable responses, and failure of therapy [15]. Stable IFD was defined as CR response to prior IFD at transplantations while active IFD was defined as PR or stable responses to prior IFD at transplantations [4]. Failure of antifungal prophylaxis was defined by any of following cases: changing to an alternative antifungal agent or dosing modification due to suspected IFD, gastrointestinal intolerance (GI) in patients with severe mucositis, colitis, diarrhea, nausea, or emesis, drug-drug interactions, persistent fever in an appropriate clinical context and failure to defervesce after administration of empiric antibiotics during IFD prophylaxis. The absence of suspected IFD, as well as no emerging IFD (for SAP patients specifically) without antifungal agent or dosing modification, were defined as successful cases.

### Administration of prophylactic antifungal agents

Antifungal prophylaxis was started on the first day of the conditioning regimen until 90 days after transplantation in patients without IFD before transplantation. SAP was given started on the first day of the conditioning regimen until 180 days after transplantation for patients with IFD before transplantation. For patients with active IFD and received SAP for more than 180 days but never reached the eradication or stability of residual foci, the SAP will be further extended till the eradication or stability of residual foci. The antifungal prophylaxis agent was given again if patients developed chronic GVHD (cGVHD), or they were treated with long-term systemic corticosteroids. Posaconazole oral suspension (200 mg orally three times a day) was administered as primary antifungal prophylaxis (PAP) and SAP for patients who had a history of IFD and achieved complete response (CR) after antifungal treatment before HSCT. Posaconazole oral suspension was administered at 400 mg twice daily as SAP for patients who had active IFD, then switched to 200 mg three times a day when the residual foci in the patients was eradicated. In case of antifungal prophylaxis failure, we would either change dose of posaconazole, or replace posaconazole with other broad-spectrum antifungal drugs (voriconazole, caspofungin, amphotericin B. or combination of voriconazole and caspofungin).

### Efficacy evaluation

All the patients were followed up until at least 12 months after allo-HSCT. IFD was routinely monitored through clinical symptoms, chest, sinuses, and/or abdomen computed tomography test, bronchoscopy and bronchoalveolar lavage serological testing for Aspergillus galactomannan, (1,3)-B-D-glucan, the culture of related tissue or samples and next-generation sequencing.

### Statistical analysis

Continuous variables were represented in median, and categorical variables were represented in percentages. The PAP and SAP groups were compared using the chi-square test for categorical variables and Wilcoxon rank sum test for quantitative data. The survival curves for OS was plotted using the Kaplan–Meier method. A multivariate analysis was performed using logistic regression for the occurrence of IFD. The cumulative incidences of prophylaxis failure, IFD by 1 year after allo-HSCT were calculated using Fine and Gray’s model with death as a competing event. As this retrospective analysis was designed as an exploratory investigation, statistical power calculation (sample size) was not conducted. The endpoint of the last follow-up for all of the surviving patients was December, 2021. SPSS16.0 (IBM, Chicago, IL, USA) was used for the statistical analysis. The R version 3.6.0 (http://www.r-project.org) software was used for competing risks analysis. P < 0.05 was considered as statistically significant.

## Results

### Patient characteristics

Among a total of 123 patients enrolled in the study, 30 (24.4%) had a past history of IFD received posaconazole as SAP and the remaining 93 (75.6%) patients received posaconazole as PAP. The patients had a median age of 39 years (range 14–72 years), with 70 (56.9%) males and 53 (43.1%) females. The underlying diseases were mainly AML (n = 41), acute lymphoid leukemia (n = 33), MDS (n = 22), aplastic anemia (n = 19), chronic myeloid leukemia (n = 4), lymphoblastic lymphoma (n = 3) and myelofibrosis (n = 1). Thirteen patients had diabetes as a complication. A hundred and three patients achieved CR and 20 patients did not the time of transplantation. 37 patients received MSD transplantation and 86 received HID transplantation. Of the 30 patients who had a history of IFD pre-transplantation, 23 patients had stable IFD and 7 patients had active IFD at the time of transplantation. Among those 30 patients, there are 5 proven IFD patients, 13 probable IFD patients and 12 possible IFD patients. We identified the pathogen in 5 proven IFD patients. Among these patients, 3 were infected by *aspergillosis* and remaining 2 were infected by *candida glabrata*. Additionally, those pathogens are theoretically sensitive to posaconazole. None of these 5 patients ever received posaconazole in their PAP treatment nor antifungal treatment. In this cohort of 123 patients, there were 21 cases (17.1%) of aGVHD (grade III-IV) and 32 (26.0%) of extensive cGVHD. Based on the patient and transplant characteristics of PAP and SAP groups in Table 1, the similar demographic and transplantation characteristics could be seen.

**Table 1:** Characteristics of patients who received posaconazole oral suspension

Characteristic	PAP (n = 93)	SAP (n = 30)	*P* value
Previous IFD			
Proven		5 (16.7)	
Probable		13 (10.6)	
Possible		12 (9.8)	
Age at transplantation (y)			0.821
Median (range)	38 (14–72)	40 (18–68)	0.258
Gender, no. (%)			0.975
Male	53 (57.0)	17 (56.7)	
Female	40 (43.0)	13 (43.3)	
Underlying disease, no. (%)			0.067
AML	30 (32.3)	11 (36.7)	
ALL	21 (22.6)	12 (40.0)	
Other^a^	42 (45.2)	7 (23.3)	
Complication, no. (%)			0.518
Diabetes	11 (11.8)	2 (6.7)	
No diabetes	82 (88.2)	28 (93.3)	
Stage of underlying disease, no. (%)			1.0
CR	78 (83.9)	25 (83.3)	
Non-CR	15 (16.1)	5 (16.7)	
Transplant type, no. (%)	10 (33.3)	20 (66.7)	0.655
MSD	27 (29.0)	10 (33.3)	
HID	66 (71.0)	20 (66.7)	
Conditioning regimen, no. (%)			1.0
Myeloablative	81 (87.1)	26 (86.7)	
Reduced intensity	12 (12.9)	4 (13.3)	
GVHD prophylaxis, no. (%)			0.394
ATG-based	75 (80.6)	22 (73.3)	
Non-ATG based	18 (19.4)	8 (26.7)	
Acute GVHD			0.624
Grades 0–II	78 (83.9)	24 (80)	
Grades III–IV	15 (16.1)	6 (20)	
Chronic GVHD			0.388
No/limited	67 (72)	24 (80)	
Extensive	26 (28)	6 (20)	
Reduced intensity	12 (12.9)	4 (13.3)	
GVHD prophylaxis, no. (%)			0.394
ATG-based	75 (80.6)	22 (73.3)	
Non-ATG based	18 (19.4)	8 (26.7)	
Acute GVHD			0.624
Grades 0–II	78 (83.9)	24 (80)	
Grades III–IV	15 (16.1)	6 (20)	
Chronic GVHD			0.388
No/limited	67 (72)	24 (80)	
Extensive	26 (28)	6 (20)	
Reduced intensity	12 (12.9)	4 (13.3)	
GVHD prophylaxis, no. (%)			0.394
ATG-based	75 (80.6)	22 (73.3)	
Non-ATG based	18 (19.4)	8 (26.7)	
Acute GVHD			0.624
Grades 0–II	78 (83.9)	24 (80)	
Grades III–IV	15 (16.1)	6 (20)	
Chronic GVHD			0.388
No/limited	67 (72)	24 (80)	
Extensive	26 (28)	6 (20)	

*PAP* primary antifungal prophylaxis, *SAP* secondary antifungal prophylaxis, *IFD* Invasive fungal diseases, *AML* acute myeloid leukemia, *ALL* acute lymphoid leukemia, Other^a^: myelodysplastic syndrome in 22 patients, aplastic anemia in 19 patients, chronic myeloid leukemia in 4 patients, lymphoblastic lymphoma in 3 patients and myelofibrosis in 1 patient. *CR* complete responses, *MSD* HLA-matched sibling donor, *HID* haploidentcal donor, *GVHD* graft-versus-host disease, *ATG* antithymocyte globulin

### Antifungal prophylaxis and efficacy

**Fig. 1:**
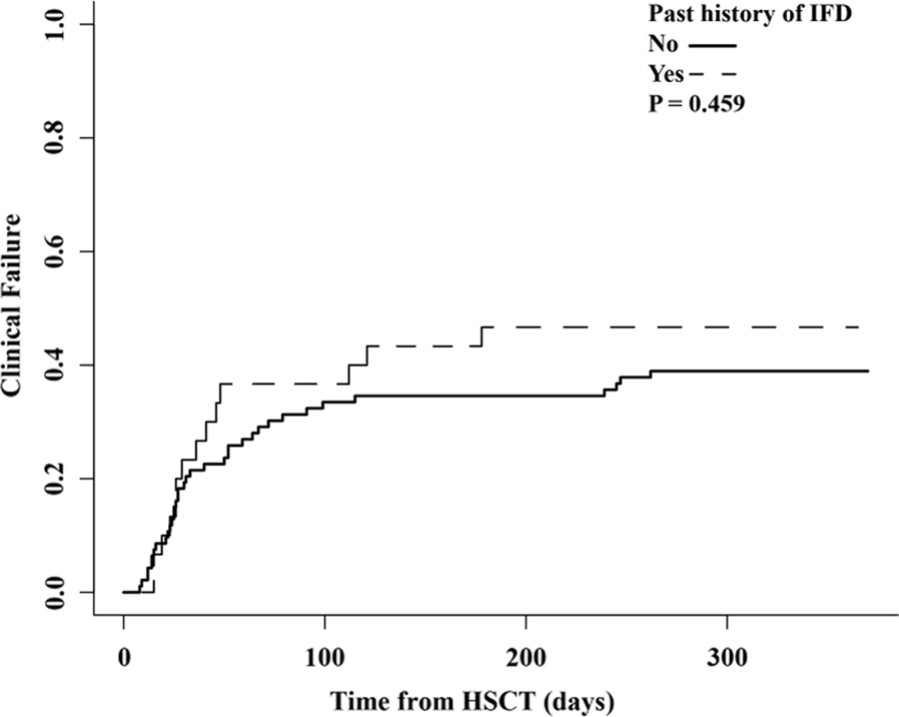
Time to clinical failure of prophylaxis

Documented reason for prophylaxis failure was identified in 43.1% (50/123) cases. There were no significant differences in the incidence of IFD, gastrointestinal intolerance, persistent fever and drug-drug interaction between the groups with a past history of IFD and the groups without. (Table 2). The median duration of antifungal prophylaxis with posaconazole was 90 days (range, 9–365 days) in PAP group and 180 days (range, 15–365 days) in SAP group. The 1-year cumulative incidence of prophylaxis failure was 58.3% (95% CI, 26.5–63.6%) in group with a past history of IFD and 41.6% (95% CI, 30.3–51.2%) in the group without a past history of IFD (p = 0.459) (Fig. 1). The primary reason for prophylaxis failure of posaconazole intervention was GI, accounting for 50.0% (25/50) of failures. It is followed by the suspected IFD, accounting for 34.0% (17/50) of failures. Five (10.0%) patients had to discontinue posaconazole due to persistent fever. Three (6.0%) patients resulted in prophylaxis failure due to drug-drug interaction that caused hepatic toxicity, but two of them were later attributed to histologically proven GVHD.

**Table 2:** Reason for failure of antifungal prophylaxis with posaconazole oral suspension

	PAP (n = 93)	SAP (n = 30)	*P*
Suspected IFD	11 (11.8)	6 (20.0)	0.259
Proven IFD	3 (3.2)	0 (0)	
Probable IFD	5 (5.4)	3 (10)	
Possible IFD	3 (3.2)	3 (10)	
Gastrointestinal intolerance	20 (21.5)	5 (16.7)	0.567
Persistent fever	3 (3.2)	2 (6.7)	0.595
Drug–drug interaction	2 (2.2)	1 (3.3)	1.0

*PAP* primary antifungal prophylaxis, *SAP* secondary antifungal prophylaxis, *IFD* Invasive fungal diseases. Suspected IFD: it includes proven/probable/possible IFDs.

### IFD occurence post-transplantation

**Fig. 2:**
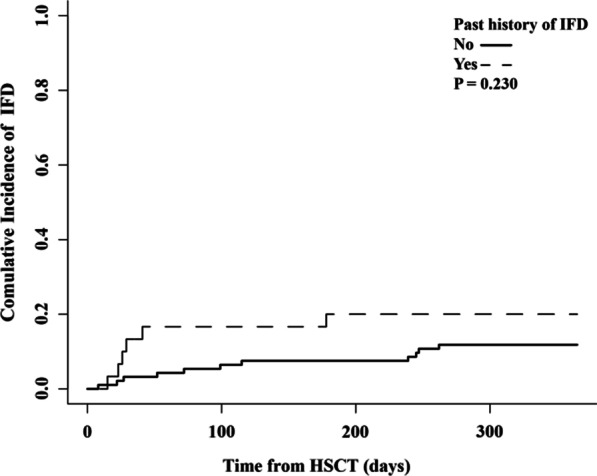
Cumulative incidence of invasive fungal diseases during the study

Among the 93 patients who used posaconazole as PAP, eleven (11.8%) cases developed IFD, including 8 during prophylaxis period and 3 after. The IFD occurred at a median time of day + 99 after transplantation (range, day + 8 to + 262). Six (20.0%) of the 30 patients with a history of IFD developed IFD during the prophylaxis. For these patients, the IFD occurred at a median time of day + 27.5 after transplantation (range, day + 15 to + 178).Two and one patient developed *aspergillosis* and *Candida spp* infection in PAP group, respectively. There were no cases of proven IFD in SAP group. Eight and 6 patients developed probable and possible IFD within 1-year after allo-HSCT, respectively. The 1-year cumulative incidence of proven, probable or possible IFD was 14.1% (95% CI, 6.0%-21.5%) in the patients without a history of IFD and 23.1% (95% CI, 5.1%-37.7%) in the group with a past history of IFD (p = 0.230, Fig. 2). The cumulative incidence of proven or probable IFD within 1-year after allo-HSCT was 10.3% (95% CI, 3.3%-16.7%) in the group without a history of IFD and 12.0% (95% CI, 0.0%-23.9%) in the group with a past history of IFD. No significant difference between those two groups was observed (p = 0.807).

### Treatment and outcome of post-transplantation IFD

Seventeen patients who developed IFD during our study received salvage therapy. Of these, eight (47.1%) patients were treated with voriconazole, 2 (11.8%) patients with caspofungin, 5 (29.4%) patients with voriconazole and caspofungin combination therapy, 1 (5.9%) patient with L-AmB and 1 (5.9%) patient with dose adjustment on posaconazole. After salvage therapy, There are 2 (11.8%) patients died of IFD, 11 (64.7%) patients achieved CR and 4 (23.5%) patients achieved PR. One (9.1%) death happened in PAP group while 1 (16.7%) happened in the SAP group.

### Risk factors for IFD

The potential risk factors for post-transplantation IFD are presented in Table 3. Having cytomegalovirus (CMV) disease post-transplantation preceding IFD [hazard ratio (HR), 12.591; 95% CI 2.571 to 61.659; *p* = 0.002] was identified as a strong risk factor for post-transplantation IFD based on both univariate and multivariate analysis.

**Table 3:** Univariate and multivariate analysis of risk factors for IFD

Risk factor	Univariate analysis		Multivariate analysis	
	HR (95% CI)	*P*	HR (95% CI)	*P*
Patient age (years)				
< 50 versus ≥ 50	0.947 (0.307–2.920)	0.925		
Gender				
Female versus male	0.503 (0.166–1.529)	0.226		
Underlying disease, no. (%)				
AML	1.0			
ALL	0.737 (0.216–2.509)	0.625		
Other^a^	0.367 (0.102–1.321)	0.125		
Stage of underlying disease				
CR versus Non-CR	0.611 (0.129–2.896)	0.534		
Diabetes				
Yes versus no	2.057(0.504–8.399)	0.315		
Transplant type				
MSD vs HID	0.543 (0.146–2.025)	0.363		
Conditioning regimen				
Reduced intensity vs Myeloablative	0.0 (0.0)	0.998		
GVHD prophylaxis				
ATG-based vs no ATG-based	0.851 (0.253–2.869)	0.795		
History of IFD pre-transplant				
No versus yes	0.537 (0.180–1.602)	0.265		
CMV DNA				
Negative versus positive	0.711 (0.245–2.067)	0.531		
CMV disease				
Yes versus no	14.306 (3.033–67.481)	0.001	12.591(2.571–61.650)	0.002
Acute GVHD				
Grades III–IV versus Grades 0–II	3.309 (1.064–10.393)	0.039	2.770 (0.801–9.579)	0.107
Chronic GVHD				
Extensive versus no/limited	1.678 (0.565–4.985)	0.351		

*IFD* Invasive fungal diseases, *AML* acute myeloid leukemia, *ALL* acute lymphoid leukemia, Other^a^: myelodysplastic syndrome in 22 patients, aplastic anemia in 19 patients, chronic myeloid leukemia in 4 patients, lymphoblastic lymphoma in 3 patients and myelofibrosis in 1 patient. *CR* complete responses, *MSD* HLA-matched sibling donor, *HID* haploidentical donor, *GVHD* graft-versus-host disease, *ATG* antithymocyte globulin

### Survival

**Fig. 3:**
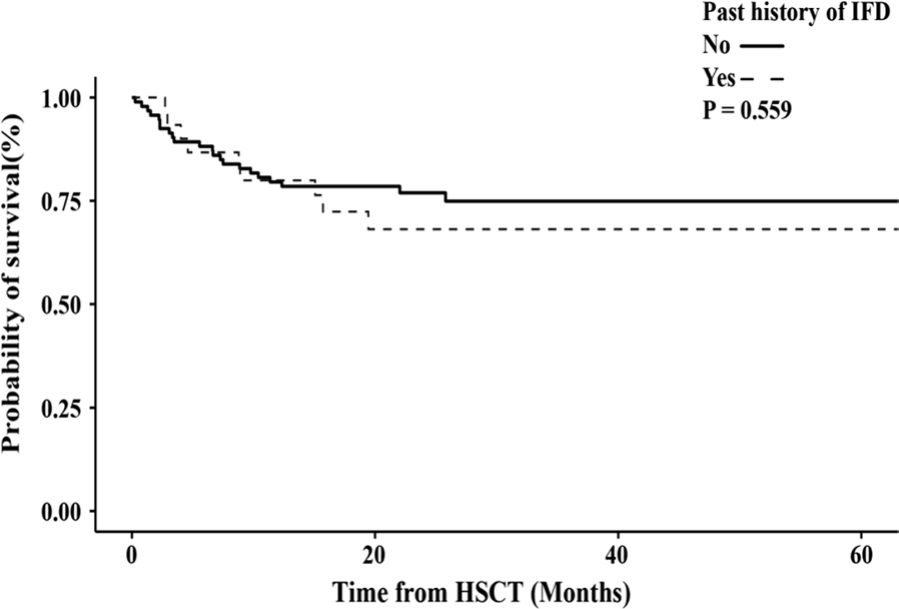
Overall survival curves in patients with or without a past history of IFD

With a median follow-up of 685 days (range 8–2005 days) post-transplantation, there were 26 deaths, the causes of which included relapse (10 cases), infections (6 cases including 2 fungal and 1 bacterial infection, 1 caused by CMV encephalitis, 1 by pneumocystis carnii and 1 by adenovirus encephalitis), GVHD (5 cases), thrombotic microangiopathy (4 cases) and hematencephalon (1 case). The 2-year OS after allo-HSCT was 74.9% ± 4.0%. No significant differences of 2-year OS were seen between PAP group and SAP group (76.9% ± 4.5% versus 68.1% ± 8.9%, *p* = 0.559; Fig. 3). In the multivariable analysis, aGVHD III-IV (HR, 5.582; 95% CI, 2.297 to 13.565; *p* = 0.001) and relapse of underlying primary diseases (HR, 3.736; 95% CI, 1.599 to 8.731; P = 0.002) were risk factors that significantly associated with poor OS (Table 4).

**Table 4:** Risk factors for overall survival

Risk factors	Univariate analysis		Multivariate analysis	
	HR (95% CI)	*P*	HR (95% CI)	*P*
Patient age (years)				
< 50 versus ≥ 50	0.687 (0.329–1.435)	0.318		
Stage of underlying disease				
NR versus CR	2.374 (1.090–5.167)	0.029	1.430 (0.541–3.778)	0.470
Transplant type				
MSD vs HID	0.876 (0.392–1.959)	0.747		
Conditioning regimen				
Reduced intensity vs Myeloablative	1.441 (0.553–3.759)	0.455		
History of IFD pre-transplant				
No versus yes	0.794 (0.366–1.724)	0.560		
CMV disease				
Yes versus no	1.663 (0.505–5.481)	0.403		
Acute GVHD				
Grades III–IV versus Grades 0–II	7.851 (3.785–16.284)	0.001	5.582 (2.297–13.565)	0.001
Chronic GVHD				
Extensive versus no/limited	0.347 (0.121–0.994)	0.049	0.421(0.141–1.258)	0.121
Post -transplantation IFD				
Yes versus no	2.082 (0.897–4.836)	0.088	1.035 (0.357–3.007)	0.949
Relapse of underlying primary diseases				
Yes versus no	6.928 (3.391–14.157)	0.001	3.736 (1.599–8.731)	0.002

*NR* no response, *CR* complete responses, *MSD* HLA-matched sibling donor, *HID* haploidentcal donor, *IFD* invasive fungal diseases, *GVHD* graft-versus-host disease

## Discussion

To our knowledge, this is the first report to evaluate the efficacy and safety of secondary prophylaxis with posaconazole in patients undergoing allo-HSCT. The number of observed prophylaxis failure (43.1%) during the study was high comparing to previous reports (33%) [9]. However, previous report mainly focused on PAP in recipients of intensive chemotherapy with a short follow-up phase [9]. Our study had found some risk factors for prophylaxis failure such as more GI events during conditioning regimens, use of corticosteroid and immunosuppressant, GVHD, CMV infections, extended follow-up time and so on. In patients with IFD history undergoing allo-HSCT, 2 retrospective studies had demonstrated the incidence of IFD was 42.9% with itraconazole as SAP agent, 31.3% with voriconazole as SAP agent, 0% to 55.5% with L-AmB as SAP agent and 13.8% to 16.7% with caspofungin as SAP agent. Our results showed a relatively low rate of the 1-year incidence of IFD (23.1%) in allo-HSCT recipients who received posaconazole as second prophylactic. It is suggested that posaconazole is relatively effective. A major cause of prophylaxis failure, accounting for nearly half of cases, was GI intolerance. Posaconazole was stopped in only three patients with abnormal liver function tests, while two of them were later found to be related to liver GVHD based on biopsy. Therefore, posaconazole is suggested to be safe and suitable for long-term treatment. The most common cause for posaconazole discontinuation was intolerance and clinician concern for absorption that occurred much earlier than discontinuations for suspected IFD, which was in line with recent reports [16]. Overall, posaconazole appears to be safe and effective in protecting such patients without severe mucositis, colitis, diarrhea, nausea, or emesis from recurring or new systemic fungal disease.

To identify the relevant risk factors of taking posaconazole orally as anti-fungal prophylaxis therapy in patients post allo-HSCT, further studies are required. CMV organ disease have been found as risk factor for IFD relapse for allo-HSCT[17]. In this retrospective study, patients with CMV organ disease also had higher incidence of IFD than those without CMV organ disease during posaconazole prophylaxis. This might be attributed to local and systemic immunosuppression induction by CMV organ disease, use of glucocorticoids, prolonged lymphopenia, GVHD and ganciclovir treatment of CMV complicated by neutropenia [17–19]. Therefore, intensification of antifungal prophylaxis and strict monitoring by radiological, serological method and molecular diagnostic should be considered for these patients [20]. However, the result should be interpreted carefully due to low amount of events in the proportional hazard model to cause inconclusive result.

It has been demonstrated that prophylaxis with posaconazole is associated with an increased efficacy of IFD prevention and an improved survival comparing to fluconazole or itraconazole prophylaxis in acute leukemia or MDS patients undergoing intensive chemotherapy [6]. Furthermore, safety and efficacy were recently assessed on a phase 3, randomized controlled, non-inferiority trial, and suggested that posaconazole had similar all-cause mortality and better tolerability than voriconazole for primary treatment in participants with invasive aspergillosis, including the patients undergoing allo-HSCT [21]. These results were similar to ours and confirmed that PAP of posaconazole was effective and safe in patients undergoing allo-HSCT. It is theoretically feasible to select the agent that had been effective, broad-spectrum and well tolerated in primary antifungal therapy for SAP. In our study, the 2-year overall survival rate of the 30 patients with IFD history was 68.1% ± 8.9% which was not significantly different than patients without a history of IFD before transplantation. The majority of deaths were caused by primary disease relapse and III–IV GVHD. This indicates that posaconazole prophylaxis can be used safely for allo-HSCT patients with a history of IFD. In addition, it is important to note that early diagnosis and prompt antifungal therapy are all critical to patient outcomes.

Our study have several limitations. First, our study is single-centered study with small sample size, which means the findings of our study may not be generalized. Second, posaconazole was switched to other antifungal agents in approximately one-fifth of patients long before discontinuations for suspected IFD. Therefore, a small portion of our results did not reflect only the effect of posaconazole prophylaxis, but also multitude of challenges in clinical practice and in a real-world in this complex patient population. Therefore, for those patients with more complexed variables should be considered. Finally, routine therapeutic drug monitoring for posaconazole was not carried out in clinical practice in this study. In the literature, the plasma concentration of posaconazole was related to the incidence of IFD [22]. This potential posaconazole serum concentration differences would make data analysis more difficult and complex, but will be considered in the future study.

## Conclusion

In summary, our study support that allo-HSCT patients without impaired GI absorption and with a prior history of IFD can choose posaconazole as prophylactic option. Our study showed posaconazole is well-tolerated over a prolonged period of time. The availability of oral posaconazole makes the agent convenient for outpatient use, for example, in patients with cGVHD or taking long-term systemic immunosuppressant. Given the limitations we mentioned above, further investigation in prospective randomized large trials is required.

## Data Availability

The datasets generated and/or analyzed during the current study are available from the corresponding author on reasonable request.The data are not publicly available due to containing information that could compromise the privacy of research participants.

## Abbreviations

allo-HSCT: Allogeneic hematopoietic stem cell transplantation
AML: Acute myeloid leukemia
ATG: Anti-thymocyte globulin
CMV: Cytomegalovirus
CR: Complete response
CsA: Cyclosporine A
GI: Gastrointestinal intolerance
GVHD: Graft-versus-host disease
HID: Haploidentical donor
HLA: Human leukocyte antigen
IFD: Invasive fungal disease
MDS: Myelodysplastic syndromes
MMF: Mycophenolate mofetil
MSD: Matched sibling donor
MTX: Methotrexate
PAP: Primary antifungal prophylaxis
PR: Partial response
SAP: Secondary antifungal prophylaxis
